# Taxonomic and Functional Responses of Soil Microbial Communities to Annual Removal of Aboveground Plant Biomass

**DOI:** 10.3389/fmicb.2018.00954

**Published:** 2018-05-31

**Authors:** Xue Guo, Xishu Zhou, Lauren Hale, Mengting Yuan, Jiajie Feng, Daliang Ning, Zhou Shi, Yujia Qin, Feifei Liu, Liyou Wu, Zhili He, Joy D. Van Nostrand, Xueduan Liu, Yiqi Luo, James M. Tiedje, Jizhong Zhou

**Affiliations:** ^1^School of Minerals Processing and Bioengineering, Central South University, Changsha, China; ^2^Institute for Environmental Genomics, University of Oklahoma, Norman, OK, United States; ^3^Department of Microbiology and Plant Biology, University of Oklahoma, Norman, OK, United States; ^4^Center for Microbial Ecology, Michigan State University, East Lansing, MI, United States; ^5^State Key Joint Laboratory of Environment Simulation and Pollution Control, School of Environment, Tsinghua University, Beijing, China; ^6^School of Civil Engineering and Environmental Sciences, University of Oklahoma, Norman, OK, United States; ^7^Earth and Environmental Science, Lawrence Berkeley National Laboratory, Berkeley, CA, United States

**Keywords:** clipping land-use, taxonomic and functional response, microbial community, metagenomics, GeoChip

## Abstract

Clipping, removal of aboveground plant biomass, is an important issue in grassland ecology. However, few studies have focused on the effect of clipping on belowground microbial communities. Using integrated metagenomic technologies, we examined the taxonomic and functional responses of soil microbial communities to annual clipping (2010–2014) in a grassland ecosystem of the Great Plains of North America. Our results indicated that clipping significantly (*P* < 0.05) increased root and microbial respiration rates. Annual temporal variation within the microbial communities was much greater than the significant changes introduced by clipping, but cumulative effects of clipping were still observed in the long-term scale. The abundances of some bacterial and fungal lineages including *Actinobacteria* and *Bacteroidetes* were significantly (*P* < 0.05) changed by clipping. Clipping significantly (*P* < 0.05) increased the abundances of labile carbon (C) degrading genes. More importantly, the abundances of recalcitrant C degrading genes were consistently and significantly (*P* < 0.05) increased by clipping in the last 2 years, which could accelerate recalcitrant C degradation and weaken long-term soil carbon stability. Furthermore, genes involved in nutrient-cycling processes including nitrogen cycling and phosphorus utilization were also significantly increased by clipping. The shifts of microbial communities were significantly correlated with soil respiration and plant productivity. Intriguingly, clipping effects on microbial function may be highly regulated by precipitation at the interannual scale. Altogether, our results illustrated the potential of soil microbial communities for increased soil organic matter decomposition under clipping land-use practices.

## Introduction

The grassland ecosystem is an important terrestrial C pool containing almost 12% of Earth’s organic matter (Schlesinger, 1977), more than 90% of which is stored belowground in the form of roots and soil organic matter (SOM) (Shahzad et al., 2012). Thus, grasslands are potential C sinks in the context of increasing global atmospheric CO_2_ concentration provided that they are properly managed (Lal et al., 2007; Li et al., 2008). Plant tissue removal via grazing, mowing and clipping plant matter, is a central issue in land-use practices and has been reported to change plant-litter decomposition (Semmartin et al., 2008; Klumpp et al., 2009), biodiversity of plants (Ward et al., 2007; Wu et al., 2009), and nutrient cycling (Garibaldi et al., 2007; de Faccio Carvalho et al., 2010). Reduced plant coverage can also change the litter layer on the soil surface, increase soil energy absorbed and emitted, and amplify the diurnal soil-temperature range (Wan et al., 2002). In addition, clipping can increase evaporation from soil and decrease transpiration of vegetation, resulting in an unpredictable net effect on soil moisture (Zhang et al., 2005).

As microbial communities play important roles in biogeochemical cycles of C, nitrogen (N), phosphorus (P), and sulfur (S), a mechanistic understanding of annual clipping effects on microbial community structure and function is crucial for a robust prediction of soil C stocks and fluxes under the context of land-use practices (Zhang et al., 2005; Belay-Tedla et al., 2009). However, until now, how microbial structure and function respond to clipping is poorly understood and remains controversial in many cases. For example, some previous studies reported that plant tissue removal can significantly reshape microbial community structure and function by increasing the ratio of oligotrophic to copiotrophic taxa (Fierer et al., 2007; Carey et al., 2015), which was mainly associated with decreased plant photosynthesis, reducing C supply to roots and belowground microbial communities (Craine et al., 1999; Bahn et al., 2006; Ingram et al., 2008). However, another study reported that little variations in microbial composition and diversity were observed under clipping treatment, although removal of aboveground plant biomass can increase soil temperature while decreasing C and nutrient pools in an experimental semi-arid grassland (Carey et al., 2015). Also, it is uncertain how plant tissue removal affects N mineralization processes and consequently alters N availability for decomposition processes (Cheng et al., 2010). In addition, interacting environmental variations such as climate (Castro et al., 2010), soil physical and chemical properties (Bell et al., 2009), vegetation (Mitchell et al., 2010), and substrate quantity and quality (Hernández and Hobbie, 2010) can significantly affect soil microbial communities, which convolute the direct impacts of plant tissue removal. Therefore, long-term monitoring of taxonomic and functional shifts of soil microbial communities in response to annual clipping is necessary for a comprehensive understanding of the effects of plant tissue removal on soil microbial communities.

The advances and applications of metagenomic technologies such as next generation sequencing and functional gene arrays (e.g., GeoChip) have revolutionized our analysis of soil microbial communities (Caporaso et al., 2012; Shokralla et al., 2012; Tu et al., 2014; Yue et al., 2015; Xue et al., 2016b). High-throughput amplicon sequencing has been successfully used to analyze the diversity of soil microbial communities in forests (Nacke et al., 2011; Brown et al., 2013; Cong et al., 2015), grasslands (Sheik et al., 2011), farmland (Su et al., 2015), and permafrost (Penton et al., 2013; Deng et al., 2015). Meanwhile, the functional gene structure and functional potentials of soil microbial communities have been rapidly analyzed using functional gene arrays, which are still quicker and less consumable for now than metagenomic shotgun sequencing especially for complex microbial communities (Liang et al., 2015; Yue et al., 2015). Therefore, the complementarity in terms of experimental data and analysis between high-throughput sequencing and functional gene arrays allows us to comprehensively estimate the composition and functional structure of soil microbial communities.

In this study, we examined taxonomic and functional responses of grassland microbial communities to annual clipping in a native, tall-grass prairie ecosystem of the US Great Plains in Central Oklahoma (latitude 34°59′ N, longitude 97°31′ W). This multifactor climate change experiment was established in 2009, with warming (+3°C), half precipitation (-50%), double precipitation (+100%), clipping (annual biomass removal) and their combined treatments (Xu et al., 2013). In this study, we primarily focus on the clipping treatment and 40 soil surface samples were collected in the clipped and control plots from 2010 to 2014 to test three central hypotheses. First, taxonomic and functional structures of soil microbial communities would be progressively altered, as the cumulative clipping effect may reduce nutrient (e.g., C, N, P) inputs from litter and change soil properties (e.g., temperature, moisture) in the long-term (5 years) scale (Hamilton and Frank, 2001; Bahn et al., 2006; Xue et al., 2016a). Also, different taxonomic and functional groups would show different sensitivities to clipping in the interannual scale due to the regulation of some temporal background variations (e.g., precipitation) on clipping effects. Lastly, clipping would significantly affect soil C and nutrient cycles by stimulating genes involved in C and N fixation and labile/recalcitrant C degradation. In this study, microbial communities were analyzed using GeoChip 5.0 as well as sequencing of bacterial/archaeal 16S rRNA gene and fungal ITS amplicons with Illumina MiSeq technology. This study provides novel insights into the taxonomic and functional responses of soil microbial communities to annual clipping and implies the potential for increased SOM decomposition under clipping land-use practices.

## Materials and Methods

### Site and Sampling

The annual clipping experiment was conducted in the Kessler Atmospheric and Ecological Field Station (KAEFS) in McClain County, OK, United States (latitude 34°59′ N, longitude 97°31′ W). KAEFS is located in the tall-grass prairie of central red-bed plains of Oklahoma, dominated by C_3_ forbs (*Ambrosia trifida, Solanum carolinense*, and *Euphorbia dentate*) and C_4_ grasses (*Tridens flavus, Sporobolus compositus*, and *Sorghum halepense*) (Xu et al., 2013). The site is on an old field prairie that had been abandoned from field cropping 40 years ago. The herbivores were excluded at this site in 2008 to prevent light grazing, which occurred before. Based on Oklahoma Climatological Survey data from 1948 to 1999, the temperature ranges from 3.3°C in January to 28.1°C in July (mean annual temperature, 16.3°C) and the precipitation ranges from 82 mm in January and February to 240 mm in May and June (mean annual precipitation, 914 mm) (Zhou et al., 2012). The soil is part of the Nash-Lucien complex with a high available water holding capacity (37%), neutral pH, and a deep (ca. 70 cm), moderately penetrable root zone (Xu et al., 2013).

This experiment was established in July of 2009 with a blocked split-plot design, in which warming (+3°C), half precipitation (-50%) and double precipitation (+100%) are primary factors nested by clipping (annual removal of above-ground biomass). The site was divided into four experimental blocks, each containing six 2.5 m × 3.5 m plots, which were further divided into two 2.5 m × 1.75 m subplots with a half for clipping. Treatments were randomly distributed across the plots within each block. Plants in the southern subplots were clipped at a height of 10 cm above the ground once to mimic the land-use practice of hay harvest at approximately the date of peak plant biomass on: 25 September, 2009; 28 September, 2010; 5 October, 2011; 17 October, 2012; 22 September, 2013; 9 October, 2014. Whereas the northern subplots were unclipped control subplots (Xu et al., 2013). The clipped plant materials were removed completely from the plots. This study focused on eight subplots with control (ambient) temperature and normal precipitation treatments, four of which were from clipped subplots and four from control (unclipped) subplots. Annual samples from the topsoil (0–15 cm) were collected one day before annual clipping from 2010 to 2014 (no samples were available in 2009). Three soil cores (2.5 cm diameter × 15 cm deep, ∼40 g) were collected in each subplot by using a soil sampler tube and composited to have enough samples for soil chemistry and molecular biology analyses. Holes were immediately refilled with root-free soils collected just adjacent to the plots. Soil samples were immediately transported to the laboratory and stored at -80°C until molecular analysis. A total of 40 annual soil samples (four clipped samples and four control samples in each year) were further analyzed in this study.

### Ambient Temperature, Precipitation, and Soil and Vegetation Property Measurements

A series of measurements were routinely performed in the experimental field. Aboveground plant biomass investigations were conducted as described previously (Sherry et al., 2008). In brief, plant biomass, separated into C_3_ and C_4_ species, was directly measured by annual clipping in the clipped subplots and indirectly estimated by the pin-contact method in the control subplots (Frank and McNaughton, 1990). Total and heterotrophic soil respirations were measured once or twice a month between 10:00 and 15:00 (local time) using a LI-8100 portable soil CO_2_ flux measurement system (LI-COR Inc., Lincoln, NE, United States), and autotrophic respiration (AR) was evaluated by the difference of total respiration and heterotrophic respiration (HR). Also, volumetric soil water content (𝜃v) from the soil surface to a 15 cm depth was measured once or twice a month using manual Time Domain Reflectometry equipment (Soil Moisture Equipment Crop., Santa Barbara, CA, United States). Three measurements of soil water content were performed in every subplot each time and the average values were used in analysis. Soil temperature was measured every 15 min at the depth of 7.5 cm in the center of every subplot, using Constantan-copper thermocouples wired to a Campbell Scientific CR10x datalogger (T-type; Campbell Science Inst., Logan, UT, United States). Air temperature and precipitation data were obtained online from an Oklahoma Mesonet Station (Washington Station) located approximately 200 m away from our experiment site. All soil samples were analyzed for soil total organic carbon (TOC) and total nitrogen (TN), soil nitrate (NO_3_^-^) and ammonia (NH_4_^+^) by the Soil, Water and Forage Analytical Laboratory at the Oklahoma State University (Stillwater, OK, United States). Soil TOC and TN concentrations were determined using a dry combustion C and N analyzer (LECO, St. Joseph, MI, United States). For NO_3_^-^ and NH_4_^+^, 6 g of soil was shaken thoroughly with 12 mL of 1 M KCl for 30 min, then filtered through a Fisher P4 qualitative filter (Fisher Scientific, Pittsburgh, PA, United States) and analyzed using a Lachat 8000 flow-injection analyzer (Lachat, Milwaukee, WI, United States). Soil pH was measured at a water-to-soil mass ratio of 2.5:1 using a pH meter with a calibrated combined glass electrode (McLean, 1982).

### DNA Extraction and GeoChip Analysis

Soil DNA was extracted from all soil samples within the same batch in 2014 by freeze-grinding and SDS-based lysis as described previously (Zhou et al., 1996), and purified by the MoBio Power Soil DNA isolation kit (MoBio Laboratories, Carlsbad, CA, United States) according to the manufacturer’s protocol. DNA quality was assessed on the basis of the ratios of 260/280 nm and 260/230 nm absorbance using a NanoDrop ND-1000 Spectrophotometer (NanoDrop Technologies Inc., Wilmington, DE, United States). The final DNA concentrations were quantified by PicoGreen using a FLUOstar Optima (BMG Labtech, Jena, Germany). The DNA samples were stored in -80°C before analyzed by the Illumina MiSeq technology (San Diego, CA, United States) and GeoChip 5.0.

The latest generation of functional gene array, GeoChip 5.0M (180K), was used to analyze the functional structure of soil microbial communities. GeoChip 5.0M contains 167,044 probes targeting 395,894 coding sequences from 1,593 functional gene families involved in C cycling, N metabolism, sulfur cycling, phosphorus cycling, electron transfer, metal homeostasis, organic remediation, stress response, secondary metabolism, and virus and virulence activity. GeoChip 5.0M was manufactured by Agilent (Agilent Technologies Inc., Santa Clara, CA, United States) in the 4 × 180K format. In our study, 800 ng of purified soil DNA of each sample was labeled with the fluorescent dye Cy-3 (GE Healthcare, Anaheim, CA, United States) using a random priming method as described previously (He et al., 2007), purified using a QIAquick Purification kit (Qiagen, Mountain View, CA, United States) according to the manufacturer’s instructions, and then dried in a SpeedVac (Thermo Savant, Holbrook, NY, United States) into powder. Subsequently, labeled DNA was resuspended into 27.5 μL of DNase/RNase-free distilled water, and then mixed completely with 99.4 μL of hybridization solution containing 63.5 μL of 2 × HI-RPM hybridization buffer, 12.7 μL of 10 × aCGH blocking agent, 10% formamide (final concentration), 0.05 μg/μL Cot-1 DNA, and 10 pM universal standard. The solution was denatured at 95°C for 3 min, and then incubated at 37°C for 30 min. Finally, the DNA solution was hybridized with GeoChip 5.0M arrays (180K) at 67°C for 24 h at 20 rpm in a hybridization oven. After hybridization, the slides were washed using Agilent hybridization buffer at room temperature and then scanned with a NimbleGen MS200 Microarray Scanner (Roche NimbleGen, Inc., Madison, WI, United States). The scanned images of the hybridized arrays were converted and extracted using Agilent Feature Extraction 11.5 software.

### GeoChip Data Processing

The microarray data were preprocessed using the microarray analysis pipeline on the Institute for Environmental Genomics (IEG) website^1^ as described previously (He et al., 2010; Tu et al., 2014). The major steps were as following: (i) Raw signal intensities (Cy3 channel) on each array were multiplied by a normalization weight I, which is the ratio of the maximum average universal standard intensity (Cy5 channel) among all the samples divided by the average universal standard intensity of each array; (ii) The signal intensities on each array were further multiplied by a normalization weight II, which is the ratio of the maximum total raw intensity (Cy3 channel) among all the samples divided by the total raw intensity of each array; (iii) Spots with SNR (signal to noise ratio) ≥ 2 were considered as positive. Otherwise they were treated as negative spots with 0 value; (iv) Spots with signal intensity lower than 250 were not considered as positive and were removed in subsequent analysis; (v) If a probe appeared in less than half or fewer of the samples in one treatment group (two out of four samples), it was removed from that group before any further analyses; (vi) The mean ratio in each sample was calculated by dividing the transformed signal intensity of each probe by the average transformed signal intensity for all detected probes in each sample. (vii) Relative change in normalized signal intensities was calculated as the clipping-induced change of gene abundance [(clipped - control)/control] in each year and/or across years.

### MiSeq Sequencing of ITS and 16S rRNA Gene Amplicons

The compositions of bacterial and fungal communities were analyzed using Illumina MiSeq sequencing of ITS and 16S rRNA gene amplicons. The V4 region of 16S rRNA genes was amplified in triplicate for each sample with the primers 515F (5′-GTGCCAGCMGCCGCGGTAA-3′) and 806R (5′-GGACTACHVGGGTWTCTAAT-3′), and ITS region was amplified in triplicate for each sample with the primers gITS7F (5′-GTGARTCATCGARTCTTTG-3′) and ITS4R (5′-TCCTCCGCTTATTGATATGC-3′). A two-step PCR was performed for ITS and 16S amplicon sequencing to avoid extra PCR bias that could be introduced by the components added in the long primers (Wu et al., 2015). The first round PCR was performed in a 25 μL reaction containing 2.5 μL 10 × PCR buffer II (including dNTPs), 0.25 U DNA polymerase, 0.4 μM of both forward and reverse target only primers and 4 μL 2 ng/μL soil DNA. Twelve cycles of PCR amplifications were performed in triplicate in the first round PCR. PCR products were purified using Agencourt^®^ Ampure^®^ XP (Beckman Coulter, Inc., Brea, CA, United States) and used as templates for the second PCR amplification of 20 cycles using the same primers, the reverse primer of which, however, contained Illumina adapter sequence and different barcodes to distinguish samples (Wu et al., 2015). The second round PCR was carried out in triplicate in a 25 μL reaction containing 2.5 μL 10 × PCR buffer II (including dNTPs), 0.25 U DNA polymerase, 0.4 μM of both forward and reverse phasing primers and 15 μL aliquot of the first round purified PCR product. PCR conditions for both first and second amplifications were as follows: 94°C for 3 min, then 94°C for 25 s, 53°C (16S rRNA gene) or 51°C (ITS) for 20 s, and 68°C for 45 s, followed by a final extension at 68°C for 10 min. PCR amplification were carried out in triplicate in order to reduce amplification bias. Subsequently, PCR products were quantified by PicoGreen using a FLUOstar Optima, combined equally and then visualized by electrophoresis on 1% agarose gels, and PCR products were purified using the QIAquick gel extraction kit (Qiangen, Valencia, CA, United States). Finally, 2 × 250 bp paired-ends DNA sequencing was performed on Illumina MiSeq platform according to the manufacturer’s instructions.

The process of sequence quality control and analysis was conducted on Galaxy pipeline^2^. Raw sequences were split to different sample libraries based on barcodes. Before the combination of forward and reverse reads, primer sequences at the end of reads were trimmed and low-quality reads were removed by the Btrim program (Kong, 2011) with threshold of QC > 20 over 5-bp window size. Forward and reverse reads of same sequence with at least 20 bp overlap and <5% mismatches were combined using FLASH program (Magoč and Salzberg, 2011). The joined sequences without ambiguous bases in length of 245–258 bp for 16S rRNA gene and 210–450 bp for ITS were subjected to chimera removal. OTUs were classified by UPARSE at 97% similarity level (Edgar, 2013), and singletons were removed. Taxonomic assignment was performed by RDP Classifier with 50% confidence estimates (Wang et al., 2007). All samples were resampled at 30,000 sequences for 16S rRNA gene and 10,000 sequences for ITS.

### Statistical Analysis

To test the significance of the differences between clipping and control treatment for various environmental variables, paired *t*-tests were employed in this study. Microbial α-diversity indexes including Shannon index, Simpson index, evenness and richness were calculated based on the three pre-processed datasets (e.g., 16S rRNA gene sequencing, ITS sequencing, GeoChip analysis). Difference of taxonomic lineages and functional genes between clipping and control was compared by the analysis of variance (ANOVA). Temporal patterns of microbial community structures in the clipped and control plots were determined by detrended correspondence analysis (DCA) based on the Bray-Curtis dissimilarity. A dissimilarity test of the microbial community structures between clipping and control was performed using non-parametric multivariate analysis of variance (Adonis) based on the Bray–Curtis dissimilarity. Mantel tests were used to calculate the correlations between environmental factors and the soil microbial communities. Canonical correspondence analysis (CCA) was performed to identify the effect of soil, plant and climate variables, and time on the microbial community structures. Based on CCA results, variation partitioning analysis (VPA) was performed to determine the contributions of each individual variable or groups of variables to total variations in the soil microbial communities. Linear and non-linear (Quadratic) models were used to reveal the correlations between environmental variables and the relative change of functional genes by clipping. All the above statistical analyses were carried out in R (v.3.1.1, The R Foundation for Statistical Computing, Vienna, Austria) using the package vegan (Dixon and Palmer, 2003).

## Results

### Ambient Temperature, Precipitation, Plant, and Soil Over Time

**FIGURE 1:**
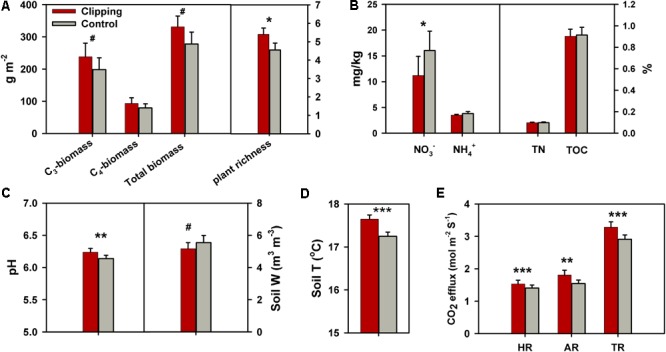
Effects of annual clipping on plant and soil variables across 5 years. **(A–E)** Effects of clipping on C_3_ plant biomass, C_4_ plant biomass, total plant biomass and plant richness **(A)**; soil nitrate (NO_3_^-^), ammonia (NH_4_^+^), total nitrogen (TN) and total organic carbon (TOC) **(B)**; soil pH and soil water content (Soil W) **(C)**; soil temperature (Soil T) **(D)**; and heterotrophic respiration (HR), autotrophic respiration (AR) and total soil respiration (TR) **(E)**. Error bars indicate standard error of the mean. The differences between clippings and controls were tested by two-tailed paired *t*-tests, indicated by ^∗∗∗^*P* < 0.01, ^∗∗^*P* < 0.05, ^∗^*P* < 0.10. The differences for some variables were also tested with one-tailed paired *t*-tests as indicated by ^#^*P* < 0.10.

During the 5 years of the experiment, the average air temperatures over autumn (September to November) were warmest in 2012 (17.2°C) and coolest in 2013 (16.4°C) (**Supplementary Figure S1a**). Autumnal cumulative precipitation ranged from 17.9 to 28.5 cm in all years except 2012 (**Supplementary Figure S1b**), which was extremely low (10.9 cm) possibly due to the most severe drought across the United States in 2012 since the Dust Bowl era of the 1930s (Wolf et al., 2016). The experimental plots were subjected to clipping once a year to mimic the land-use practice of hay harvest since 2009. The total plant biomass across 5 years (2010–2014) was marginally significantly (*P* = 0.06) increased under clipping (**Figure 1A**), based on the one-tailed paired *t*-test. Specifically, clipping did not significantly change total plant biomass in the first 3 years (2010–2012), but significantly increased total plant biomass in 2013 (*P* = 0.03) and 2014 (*P* = 0.05) (**Supplementary Table S1**). The C_4_ plant biomass was significantly (*P* = 0.02) higher under clipping than control, but the C_3_ plant biomass remained unchanged in 2014 (**Supplementary Table S1**), resulting in a plant community shift toward more C_4_ plant species. In addition, plant richness was marginally significantly (*P* = 0.06) increased by 5 years of clipping (**Figure 1A**).

Soil process measurements revealed that overall soil TOC, TN, and soil ammonium-N (NH_4_^+^-N) remained unchanged under annual clipping (**Figure 1B**). However, the concentrations of NO_3_^-^-N were marginally significantly (*P* = 0.06) decreased by 5 years of clipping. Specifically, NO_3_^-^ became significantly (*P* < 0.05) lower under clipping than control from 2012 to 2014 (**Supplementary Table S1**). Furthermore, soil pH was also significantly (*P* = 0.03) increased by annual clipping (**Figure 1C**), while annual clipping marginally significantly decreased soil water content based on a one-tailed paired *t*-test. In addition, the average temperature in the surface soil (top 15 cm) significantly (*P* < 0.01) increased under clipping in each year, resulting in an increase of 0.4°C across 5 years (**Figure 1D**). Also, plant belowground activity and microbial activity, measured as total soil respiration (TR), HR, and AR, were significantly (*P* < 0.05) higher in clipped plots than control plots. Annual clipping significantly (*P* < 0.01) increased the rates of AR and HR by 17% and 9%, respectively, suggesting that annual clipping stimulated the activities of plant roots and microbial SOM decomposition simultaneously (**Figure 1E**).

### Overall Responses of Soil Microbial Communities to Annual Clipping

Soil microbial communities were analyzed by sequencing 16S rRNA gene and ITS amplicons with Illumina Miseq and functional gene arrays (GeoChip 5.0M). The non-parametric multivariate analysis of variance revealed that taxonomic and functional structures of microbial communities were much more strongly influenced by annual temporal variation (explaining 16.9–48.1%) than annual clipping (explaining 2.5–2.8%) (**Table 1**). No significant differences were observed in the overall bacterial and fungal diversities and structures between clipped and control samples in all years (**Table 1, Supplementary Figures S2a,b**, and **Supplementary Table S2**). One exception to this was bacterial community in 2012, which had significantly fewer OTUs under clipping than control. Further comparison of the microbial taxonomic composition showed that some key bacterial and fungal phyla were significantly (*P* < 0.05) shifted by annual clipping (**Supplementary Figures S3, S4**). Specifically, *Actinobacteria, Bacteroidetes, Crenarchaeota*, and *Gammaproteobacteria* were significantly (*P* < 0.05) decreased by 5 years of clipping, and *Chloroflexi* and *Planctomycetes* in bacterial community were significantly (*P* < 0.05) increased by 5 years of clipping (**Supplementary Figure S3**). In fungal community, the phyla *Zygomycota* and *Ascomycota* were significantly (*P* < 0.05) decreased under annual clipping across 5 years (**Supplementary Figure S4**). However, different phyla and genera in bacterial and fungal communities showed greatly different sensitivities to clipping in different years, as indicated that different phyla and genera were significantly (*P* < 0.05) or marginally significantly (*P* < 0.10) shifted by clipping in different years (**Supplementary Table S3**). Among these years, significantly and marginally significantly changed bacterial and fungal genera were the most in 2012, most of which belonged to *Actinobacteria* (16 genera), *Alphaproteobacteria* (15 genera), *Bacteroidetes* (9 genera), and *Ascomycota* (6 genera). Intriguingly, the relative abundances of unidentified fungi were greatly increased in 2012, and the relative abundance of unidentified fungi was significantly (*P* < 0.05) higher under clipping (49.5%) than control (13.7%) (**Supplementary Figure S4** and **Supplementary Table S3**).

**Table 1:** Significance tests of the effects of clipping and year on the overall microbial community structures across 5 years and in each year by the non-parametric multivariate analysis of variance.

	16S rRNA		ITS		GeoChip	
	*R*^2^	*P*	*R*^2^	*P*	*R*^2^	*P*
Year	0.169	**0.002**	0.183	**0.001**	0.481	**0.001**
Clipping	0.025	0.352	0.028	0.179	0.028	**0.020**
Year × clipping	0.103	0.208	0.096	0.313	0.228	**0.001**
2010 clipping	0.178	0.183	0.176	0.287	0.291	0.115
2011 clipping	0.143	0.506	0.142	0.434	0.306	**0.026**
2012 clipping	0.178	0.210	0.221	0.058	0.731	**0.034**
2013 clipping	0.125	0.632	0.121	0.855	0.256	**0.049**
2014 clipping	0.132	0.605	0.110	0.756	0.241	**0.027**

*Bold values indicate *P* < 0.05.*

Annual clipping significantly shifted the functional gene richness and diversity, measured as the number of functional genes, Shannon diversity, Simpson diversity and evenness (**Supplementary Table S2**). There were marginally (*P* < 0.10) or significantly (*P* < 0.05) more functional genes detected in clipped samples than control samples in 2011, 2013 and 2014. However, the numbers of functional genes detected and Shannon diversity in 2010 and 2012 were significantly (*P* < 0.05) lower in clipped plots than those in control plots. More importantly, annual clipping also significantly (*P* < 0.05) changed the functional structure of microbial community (**Table 1**). The non-parametric multivariate analysis of variance in each year revealed that no significant clipping effect was observed in the first year (2010), but clipping effects became significant (*P* < 0.05) in the following 4 years (2011–2014) (**Table 1**). These results indicated that the shifts in microbial community functional structure under annual clipping progressively deepened along time and that annual clipping had cumulative effects on microbial community over time. DCA showed that clipped and control samples were clustered together in the first year (2010), while clipped samples gradually separated by the first DCA axis from control samples in the following 4 years (2011–2014) (**Supplementary Figure S2c**). Furthermore, the shifts in microbial community function under clipping were consistent in direction of the first DCA axis in 2011, 2013, and 2014. Unexpectedly, the shift in microbial community function under clipping in 2012 was abnormally bigger than those in the other years and opposite in directionality, possibly due to the strong perturbance of the other environmental factors or the infestation of unidentified fungi.

### Linking Microbial Communities to Environmental Variables

**FIGURE 2:**
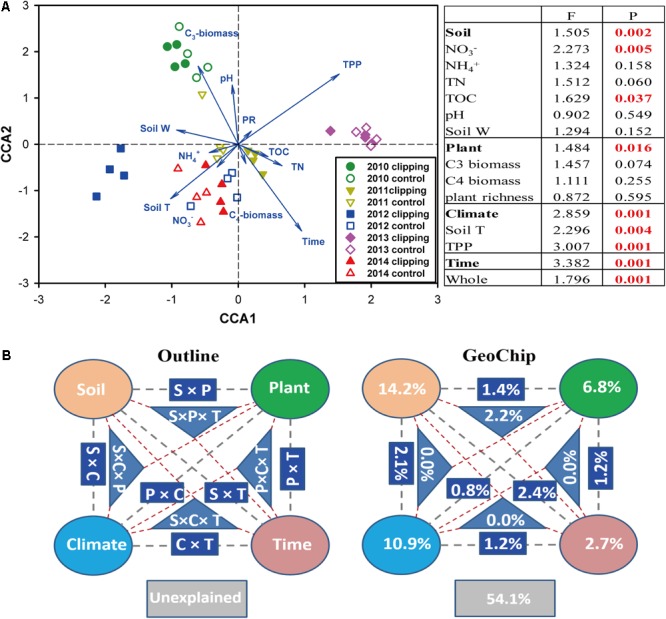
Constrained ordination analysis. **(A)** Canonical correspondence analysis (CCA) of GeoChip data and environmental variables. environmental variables: soil nitrate (NO_3_^-^), ammonia (NH_4_^+^), total organic carbon (TOC), total nitrogen (TN), soil pH, soil water content (soil W), aboveground C_3_ biomass, C_4_ biomass, plant richness (PR), soil temperature (Tm) and autumnal total precipitation (TPP). The insert table showed the significances of each or subsets of the environmental variables in explaining the variations of microbial community functional structure based on *F*-test. **(B)** CCA-based variation partitioning analysis (VPA) of microbial functional structure explained by soil geochemical properties (S), plant diversity (P), climate (C) variables and time (T). Each diagram represents the biological variation partitioned into the relative effects of each factor or a group of factors.

Canonical correspondence analysis and Mantel test were performed to discern the linkage between soil microbial phylogenetic and functional structures and environmental factors (**Figure 2** and **Supplementary Figures S5, S6**). The CCA results indicated that microbial functional structure was significantly (*F* = 1.796, *P* = 0.001) shaped by several soil, plant and climate variables as well as time (**Figure 2A**). Among these factors, time, precipitation, temperature and C_3_ biomass exhibited more significant (*P* < 0.05) correlations with the variations of microbial functional structure. These soil, plant, climate variables and time exhibited significant correlations with taxonomic structure of bacterial community (*F* = 1.135, *P* = 0.026) but not fungal community (*F* = 0.966, *P* = 0.746) based on the CCA results (**Supplementary Figures S5a, S6a**). Furthermore, several key plant and soil variables also exhibited strong correlations with both functional and taxonomic community structures by Mantel tests (**Table 2**). For example, C_3_ biomass and total biomass showed significant (*P* < 0.05) correlation with bacterial, fungal and functional community structures, and soil temperature showed significant (*P* < 0.05) correlation with bacterial and functional community structures. Importantly, HR exhibited significant correlations with bacterial community (*P* = 0.076), fungal community (*P* = 0.045), and microbial functional structure (*P* = 0.022) as revealed by Mantel tests (**Table 2**). These results indicated that the shifts of microbial communities were significantly (*P* < 0.05) correlated with clipping-induced changes of soil microclimate, soil respiration and aboveground plant productivity.

**Table 2:** Correlation analysis between microbial community structures and environmental variables by Mantel test^a^.

environmental variables	16S rRNA		ITS		GeoChip	
	*r*	*P*	*r*	*P*	*r*	*P*
All variables	0.215	**0.015**	0.179	**0.034**	0.107	**0.099**
Time	0.136	**0.019**	0.295	**0.001**	0.222	**0.001**
NO_3_^-^-N	0.049	0.286	–0.023	0.563	–0.082	0.791
NH_4_^+^-N	0.054	0.261	0.164	**0.047**	–0.111	0.927
TN	0.022	0.371	0.109	0.125	–0.106	0.939
TOC	0.032	0.336	0.107	**0.089**	–0.124	0.970
Soil pH	0.150	**0.026**	0.044	0.283	0.138	**0.047**
Soil T	0.116	**0.040**	0.046	0.243	0.101	**0.049**
Soil W	0.002	0.474	0.110	0.102	0.084	0.139
C_3_ biomass	0.145	**0.036**	0.288	**0.001**	0.224	**0.005**
C_4_ biomass	0.024	0.359	0.097	0.111	–0.002	0.462
Total biomass	0.133	**0.044**	0.237	**0.010**	0.215	**0.009**
Plant richness	–0.065	0.821	0.114	**0.052**	–0.038	0.679
Precipitation	0.042	0.190	0.091	**0.037**	0.201	**0.002**
HR	0.107	**0.076**	0.120	**0.045**	0.135	**0.022**
TR	0.100	0.163	–0.003	0.505	0.053	0.263

*^*a*^The significant value (*P* < 0.10) are indicated in bold. NO_*3*_^-^-N, soil nitrate-nitrogen; NH_*4*_^+^-N, soil ammonium nitrogen; TN, soil total nitrogen; TOC, soil total organic carbon; soil W, soil water content; soil T, soil temperature; HR, soil heterotrophic respiration; AR, soil autotrophic respiration; TR, soil total respiration.*

**FIGURE 3:**
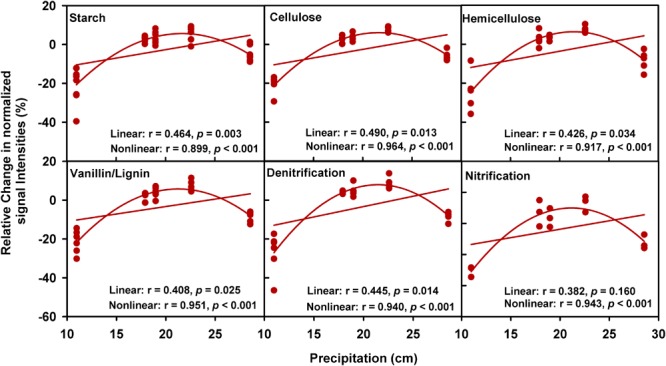
Relationships between autumnal cumulative precipitation and clipping effects on C-degrading and N cycling gene groups. For all plots, the relative change of normalized signal intensities of detected genes by clipping in each year was presented in the *y*- axis as the clipping effects on functional genes [(clipping – control)/control]. In each plot, lines represent linear least squares regression fit and non-linear quadratic regression fit, respectively. The *r* values and significances were displayed for linear and non-linear fits. The genes in these plots are listed in **Supplementary Table S4**.

A partial CCA-based VPA indicated that these soil, plant, climate variables as well as time could explain more variations based on GeoChip data (42.4%, **Figure 2B**) than 16S rRNA gene (30.4%, **Supplementary Figure S5b**) and ITS (27.2%, **Supplementary Figure S6b**) sequencing data, suggesting that functional structure of microbial communities is more sensitive to detect clipping-induced environmental changes than taxonomic structure of microbial communities. Specifically, the variations in the community functional composition and structure were explained by soil (14.2%) and plant (6.8%) variables, time (2.7%) and their interactions (7.2%; **Figure 2B**). Soil temperature and precipitation alone could directly explain 10.9% of the variation in community functional structure (**Figure 2B**). These results indicated that temperature and precipitation were important environmental attributes that shape the microbial community under clipping treatment. Furthermore, clipping effects on some C-degrading and nitrogen cycling genes were significantly (*P* < 0.05) regulated by autumnal cumulative precipitation, as indicated that clipping-induced changes in some key genes for C degradation and N cycling were linearly (*P* < 0.05) or non-linearly (*P* < 0.05) increased along precipitation, including those for degrading starch, cellulose, hemicellulose and Vanillin/Lignin, denitrification and nitrification (**Figure 3**). Clipping effects on these genes in the driest year 2012 were substantially different from those in the other years (**Figure 3**). Therefore, the response of functional genes to clipping in 2012 possibly represented a feedback pattern under the extreme drought condition, which was greatly different from the long-term pattern of microbial functional changes under annual clipping.

### Effects of Annual Clipping on Microbial Functional Genes

**FIGURE 4:**
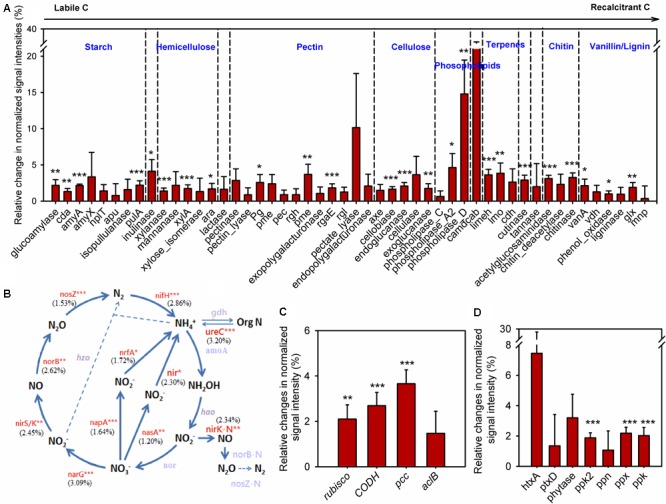
Clipping effects on functional genes involved in biogeochemical cycling processes. **(A)** C degradation. The complexity of C is presented in order from labile to recalcitrant C. The average relative change of normalized signal intensity of detected genes by clipping across 5 years without 2012 samples was presented as the clipping effects on functional genes [(clipped – control)/control]. Error bars indicate standard error of the mean. Significance was tested by ANOVA as indicated by ^∗∗∗^*P* < 0.001, ^∗∗^*P* < 0.01, ^∗^*P* < 0.05. **(B)** N cycling processes. The percentage changes in N cycling gene abundance under clipping were indicated in parenthesis. Genes where change in abundance was significant (*P* < 0.05) are labeled in red. Gray-colored genes were not significantly changed under clipping. **(C)** C fixation. **(D)** P utilization genes under annual clipping. The full names of the genes in this figure are listed in **Supplementary Table S4**.

To understand how annual clipping affected functional processes of soil microbial communities, GeoChip data were further analyzed by focusing on C, N, and P cycling. The normalized signal intensities were calculated to evaluate the change of gene abundance under clipping in each year. Because the shift in microbial communities under clipping in 2012 appeared in stark opposition to all other years and 2012 correspondingly was a year with prolonged and wide-spread drought (Cook et al., 2014; Wolf et al., 2016), average normalized signal intensities across 5 years with the omission of the year 2012 were evaluated to obtain the long-term trends of C, N, and P cycling under annual clipping (**Figure 4**).

In the first year (2010), most C degradation genes were significantly (*P* < 0.05) decreased under clipping (**Supplementary Figure S7**). However, clipping increased the abundance of most C degradation genes in the second year (2011). Among these genes, 17 genes whose abundance significantly (*P* < 0.05) increased under clipping were those involved in the degradation of relatively labile C (e.g., starch, hemicellulose, pectin, and cellulose). For example, glucoamylase, involved in the degradation of starch, xylanase, which degrades hemicellulose, and cellobiase, which breaks down cellulose, all showed significantly (*P* < 0.05) higher signal intensities under clipping. Interestingly, clipping also significantly (*P* < 0.05) increased the abundance of five genes involved in the degradation of recalcitrant C (e.g., chitin, vanillin, and lignin) including those encoding chitinase and phenol oxidase (**Supplementary Figure S7**). In contrast, in 2012, the year with low precipitation, almost all of detected C degradation genes decreased in the relative abundance under clipping. In the last 2 years (2013 and 2014), very few genes associated with labile C degradation remained significantly increased under clipping, while most of the genes involved in recalcitrant C degradation, that had originally significantly increased in 2011, were again significantly (*P* < 0.05) increased under clipping (**Supplementary Figure S7**). These results suggested that the stimulation of clipping on the genes involved in recalcitrant C degradation was more persistent than genes involved in labile C degradation, and implied that the degradation of recalcitrant C might be triggered as the cumulative effect of annual clipping on microbial communities increased over time. Furthermore, the average signal intensities across 5 years with the omission of the third year (year 2012) also indicated that annual clipping significantly (*P* < 0.05) increased the relative abundances of many genes involved in the degradation of labile and recalcitrant C (**Figure 4A**).

GeoChip 5.0M also has various probes for key enzymes in CO_2_ fixation from four pathways: ribulose-1, 5-bisphosphate carboxylase/oxygenase (Rubisco) in the Calvin cycle, propionyl-CoA carboxylase (PCC) in the 3-hydroxypropionate cycle, carbon monoxide dehydrogenase (CODH) in the reductive acetyl-CoA pathway and ATP citrate lyase (AclB) in the reverse tricarboxylic acid cycle. All of these key genes fluctuated greatly in different years, likely reflecting the interaction of annual clipping and annual temporal variation over time (**Supplementary Figure S8**). In 2010 the abundances of *rubisco, codh*, and *ppc* genes were significantly (*P* < 0.05) decreased under clipping, but these genes significantly (*P* < 0.05) increased under clipping in 2011. In the last 2 years, *rubisco* genes (2013 and 2014), *codh* genes (2014), and *ppc* genes (2014) were significantly increased under clipping (**Supplementary Figure S8**). The year of 2012 represents a unique set of environmental conditions and a strong response shift to clipping treatment by the microbial community was observed. In 2012, all of the key genes in C fixation incongruently decreased under clipping. However, the average signal intensities across 5 years without 2012 indicated that the abundances of *rubisco, codh*, and *ppc* genes were significantly (*P* < 0.05) increased under annual clipping (**Figure 4C**). These results suggested that CO_2_ fixation might be enhanced under 5-years of clipping treatment, but further studies are needed to determine the impacts of the fixed C on the overall soil carbon flux.

The abundances of N cycling genes involved in ammonification, anammox, assimilatory N reduction, denitrification, dissimilatory N reduction, N assimilation, nitrification, and nitrogen fixation were also shifted in different years (**Supplementary Figure S9**). The relative changes of average signal intensities of these years without 2012 were also analyzed to determine the long-term trend of N cycling under clipping. The abundance of most key genes involved in N cycling was significantly (*P* < 0.05) higher under annual clipping than control (**Figure 4B**). Specifically, the gene *ureC* and ammonium transporter gene (*gdh*) were significantly (*P* < 0.05) increased in clipped samples. Because the UreC protein can convert urea into NH_4_^+^, and ammonium transporter proteins transport ammonium into microorganisms or plants, the combined effect of these two changes could potentially result in an increase in N mineralization but relatively stable NH_4_^+^ concentrations in the soil. Furthermore, *nirB* and *nasA*, involved in assimilatory N reduction, *norB, nirK, nirS*, and *narG* associated with denitrification, *napA* a dissimilatory N reductase, were also significantly (*P* < 0.05) increased under clipping treatment (**Figure 4B**). The combined effect of denitrification, assimilatory N reduction and dissimilatory N reduction could result in a rapid nitrate-nitrogen loss, which may be the reason why significantly decreased NO_3_^-^ in soil were observed under clipping in 2012–2014 (**Supplementary Table S1**). In addition, the relative abundance of *nifH* for nitrogen fixation was significantly (*P* < 0.05) increased under 5-year clipping (**Figure 4B**). These significantly increased genes in nitrogen-cycling process may potentially lead to accelerating nutrient cycling rates under annual clipping.

GeoChip 5.0M contains seven enzymes involved in P utilization; exopolyphosphatase (Ppx), endopolyphosphatase (Ppn), and polyphosphate kinase (Ppk2) involved in phosphate degradation, polyphosphate kinase (Ppk) in polyphosphate biosynthesis pathways in prokaryotes, phosphonate dehydrogenase (PtxD) and phytanoyl-CoA dioxygenase (HtxA) responsible for phosphorus oxidation, and phytase associated with phytate degradation. All of these genes were also shifted in different years (**Supplementary Figure S10**). The abundance of *ppk*2 and *ppx* genes across 5 years, without 2012, were significantly (*P* < 0.05) increased under clipping, suggesting a possible increase of polyphosphate degradation with more available inorganic P in soil under annual clipping (**Figure 3**). Also, the abundance of *ppk* in clipped samples was significantly (*P* < 0.05) higher than in control samples (**Figure 4D**). Altogether, this indicated that P cycling potentials under annual clipping might also be accelerated in this ecosystem.

## Discussion

Plant tissue removal via grazing, mowing and/or clipping can significantly change ecosystem C fluxes, with consequent changes in plant-litter decomposition, soil microbial communities and nutrient cycling (Garibaldi et al., 2007; Klumpp et al., 2009; de Faccio Carvalho et al., 2010). As soil microbial community mediate important biogeochemical processes, such as C, N, P, and S cycling, understanding their responses to annual clipping is crucial for a robust prediction of soil C stocks and fluxes. In this study, we analyzed the potential taxonomic and functional responses of soil microbial communities to annual clipping. Our results showed that annual clipping markedly shifted the functional structures of soil microbial communities and relative abundances of some bacterial and fungal lineages over time, which generally support our three hypotheses.

Previous studies showed that clipping significantly affected the composition and productivity of plant communities (Ward et al., 2007; Wu et al., 2009), likely decreasing nutrient and C inputs from aboveground plants (Semmartin et al., 2008; Klumpp et al., 2009). Furthermore, clipping has been shown to increase soil temperature but decrease soil water content as did warming (Wan et al., 2002). Under clipping, an increase of root respiration and exudation was also observed (Bahn et al., 2006; Hamilton et al., 2008). Collectively, these shifts in plants and microenvironments under the cumulative effects of clipping are expected to progressively affect the structure and functional potential of soil microbial communities across a time span of several years. Our results generally supported this hypothesis. Consistent with those previous studies, we found annual clipping increased plant productivity, soil CO_2_ efflux and microbial activity in most of years. More importantly, the microbial functional structure was not changed in the first year but altered significantly in the following 4 years by annual clipping (**Table 1**). Furthermore, statistical analyses proved that the changes of microbial community structure were significantly correlated with soil respiration, soil physical and chemical variables, and above-ground plant productivity. Besides, the relative abundance of some key bacterial and fungal phyla such as *Actinobacteria, Bacteroidetes, Zygomycota*, and *Ascomycota* were significantly altered across 5 years by annual clipping. These results demonstrated that the shifts of soil microbial communities under a long-term clipping can cumulatively affect certain soil ecosystem functions. However, certain studies suggested clipping or mowing reduced soil CO_2_ efflux, microbial biomass and activity due to decreased canopy photosynthesis and lessened C supply from aboveground plant parts to roots, mycorrhizae and rhizosphere microorganisms (Zhang et al., 2005; Bahn et al., 2006; Shahzad et al., 2012). The disparity among studies may be caused by the different ecosystems studied and/or experimental designs including clipping and sampling time.

Microbial responses to global changes, such as warming, precipitation, and clipping may be greatly regulated by temporal background variations. Previous studies at the Jasper Ridge Global Change Experiment (JRGCE) showed that annual background variation of soil microbial communities was greater than even very significant treatment effects including warming, elevated CO_2_, water addition, and N addition (Gutknecht et al., 2012). Another study also reported that temporal (seasonal and interannual) variation overshadows the responses of soil microbial communities to simulated global changes including drought and N addition (Matulich et al., 2015). Consistently, the taxonomic composition of both bacterial and fungal communities varied substantially from year to year in our study. No significant clipping effect was observed in the overall bacterial and fungal communities, most likely due to the large interannual background variation in soil microbial community overshadowing the response of bacterial and fungal communities to clipping (Matulich et al., 2015). Correspondingly, interannual background variations affected the relative abundance of bacterial and fungal phyla more significantly than annual clipping. Also, interannual background variation was more significant than the effect of clipping on soil microbial functional genes. The abundance of many functional genes involved in C fixation, C degradation, N cycling, and P utilization greatly fluctuated in different years. These results suggested that microbial responses to annual clipping were strongly shaped by temporal background variations.

More interestingly, we found a stark contrast in the functional community response to clipping when the ecosystem underwent an extreme drought disturbance as well as a significant correlation between precipitation and clipping-induced changes in some C-degrading or N cycling genes. Furthermore, precipitation was found to be one of the most important factors in explaining the variations of functional community structure in our study. Previous studies also reported that altered precipitation in different years can significantly change fungal and bacterial community structures (Schmidt et al., 2007; Castro et al., 2010). Precipitation can shift microbial biomass, community composition and activity directly by changing soil moisture as well as indirectly through shaping plant community, potentially with a lag (Schmidt et al., 2007; Castro et al., 2010). Provided these information, it may be that the effects of clipping on microbial functional activities are strongly associated with precipitation at the interannual scale.

In our study, no significant changes of the overall taxonomic structure of bacteria and fungi were observed in all years, whereas microbial functional structure was significantly shifted by clipping in the continuous four years. Furthermore, the variations of soil microbial functional structure were closely related to clipping-induced environmental changes, while the taxonomic variations were only poorly explained by environmental condition. No significant correlation was observed between taxonomic groups and functional gene groups. Such phenomena have been previously observed in the global ocean or in soil (Raes et al., 2011; Louca et al., 2016; Nelson et al., 2016). These results can be explained by an elegant paradigm for microbial ecology, in which community function is strongly shaped by energetic and stoichiometric constraints (Raes et al., 2011), while the composition within functional groups is modulated by additional mechanisms. According to this paradigm, the functional responses of microbial communities to clipping can decouple with microbial taxonomic variations.

Whether the clipping-stimulated microbial community resulted in the significant changes of soil C and N cycling is another central hypothesis. Some studies showed that clipping reduced total soil CO_2_ efflux composing of root respiration and mineralization of plant litter and recalcitrant SOM by 20–50% (Wan and Luo, 2003; Shahzad et al., 2012). In contrast, another study showed a TR increase under clipping treatment (Antonsen and Olsson, 2005). Significant increases in total and heterotrophic soil respirations by clipping were observed in our study. Theoretically, the increase of soil respiration could be due to the increase of microbial biomass and/or the variation of microbial community structure (Marschner et al., 2015; Bond-Lamberty et al., 2016). However, very limited amounts of samples were available from this long-term field experiment, thus we didn’t investigate microbial biomass. In this study, we focused on the variation of community structure. Significant correlations between soil HR and the variations of bacterial, fungal and functional community structures suggested that the variation of community structure significantly modified soil C cycling, regardless of potential altered microbial biomass.

Although annual background variations (e.g., precipitation) strongly affected functional patterns of soil microbial community in different years, progressive changes of C degradation under clipping were still observed in our GeoChip data. In the first year, annual clipping as a strong disturbance to grassland ecosystem not only decreased soil microbial community functional diversity but also decreased most of gene abundances involved in C degradation. This may be a short-term response to the decrease of aboveground C input and the sudden changes of soil temperature and moisture under annual clipping. In the following 4 years, except 2012, the abundance of key genes involved in the degradation of labile and recalcitrant C increased under annual clipping, suggesting that reduced inputs of aboveground C under clipping did not suppress microbial activity, probably because of the offset by elevated belowground biomass through such processes as root exudation (Hamilton et al., 2008) and root decay (Belay-Tedla et al., 2009). There were no significant reductions in aboveground plant biomass in the clipped plots and, in fact, in the last 2 years there were significant increases in the clipped plots. This means that the plant growth rates were stimulated by annual clipping. A likely consequence of this, is enhanced root development and increased exudation by actively growing roots. Indeed, significant increase of root respiration under clipping, measured as AR, was observed in this grassland ecosystem. This may be the reason why soil total C did not significantly decrease under annual clipping. More importantly, the abundances of genes involved in the degradation of some recalcitrant substrates were consistently increased in the last 2 years under annual clipping, indicating that the recalcitrant C degradation may be triggered under annual clipping. Since the recalcitrant carbon in soil is much more abundant than labile carbon, even a small change in its decomposition rate could have significant effect on soil C storage (Davidson and Janssens, 2006). By this way, clipping land use practices may significantly affect future climate warming scenarios.

How clipping or mowing changes ecosystem N cycling is another important issue. A previous study showed that total N contents of soil at the Great Plains Apiaries, Oklahoma were significantly decreased by clipping, resulting in N-deficient soil conditions (Belay-Tedla et al., 2009), and another study showed that yearly clipping significantly decreased litter N contents, indicating a significant effect of N deficiency on plants (Cheng et al., 2010). Consistently, a significant decrease in NO_3_^-^-N was observed under annual clipping in our study, suggesting that soil N dynamics were significantly altered. In our GeoChip data, average signal intensities across 5 years with the omission of the year 2012 indicated that annual clipping also stimulated the abundance of most key genes involved in N cycling, including ammonification, denitrification, N assimilation, and nitrogen fixation. The significant increases in the abundance of N cycling genes may result in a potential increase of nutrient cycling process rates. In high N cycling rates, N fixation and N mineralization through recycling N from SOM would compensate N loss by denitrification as well as enhance plant growth (Zhou et al., 2012). As a result, the total soil N may remain unchanged under annual clipping. However, the effects of long-term clipping on soil N dynamics may depend on the balance of the accumulation derived from the inputs from litter and root biomass decomposition, microbial N fixation and the consumption of N mineralization, denitrification, and plant uptake.

## Conclusion

Despite the important roles of the soil microbial communities in carbon and nitrogen cycling, the responses of microbial community structure and function under long-term clipping are not fully understood. In this study, the functional structure of soil microbial community was significantly altered by 5 years of clipping and the relative abundance of bacterial and fungal lineages was also significantly changed under annual clipping. Furthermore, annual clipping significantly increased the abundance of genes involved in the degradation of labile and recalcitrant C, nitrogen cycling and phosphorus utilization in the long-term scale. The shifts in microbial community structure and function were significantly correlated with soil microclimate, C and nutrient concentrations, respiration and plant productivity. Interestingly, the effects of clipping on microbial functional activities may be heavily associated with precipitation at the interannual scale. Annual clipping-induced changes in microbial community structure and function may be important in predicting long-term land-use responses to global change.

## Data Accessibility

DNA sequences of 16S rRNA gene and ITS amplicons were deposited under NCBI project accession no. PRJNA331185. OTU table and OTU representative sequences are available: http://ieg.ou.edu/4download/. Microarray data (GeoChip 5.0) are available: http://ieg.ou.edu/4download/. Soil physical and chemical attributes, plant biomass and richness and soil respirations: online Supplementary Materials.

## Author Contributions

All authors contributed intellectual input and assistance to this study. The original concept and experimental strategy were developed by JZ, YL, and JT. Field management was carried out by MY, JF, XG, XZ, LH, FL, LW, and JVN. Sampling collections, DNA preparation, and MiSeq sequencing analysis were carried out by XZ, XG, JF, MY, and LH. Soil chemical analysis was carried out by XZ, XG, and MY. Various statistical analyses were carried out by XG, YQ, DN, and ZS. Assistance in data interpretation was provided by XL and ZH. All data analysis and integration were guided by JZ. The paper was written by XG with help from ZH and JZ. Considering their contributions in terms of site management, data collection, analyses, and/or integration over the last 6 years, XG and XZ were listed as co-authors.

## Conflict of Interest Statement

The authors declare that the research was conducted in the absence of any commercial or financial relationships that could be construed as a potential conflict of interest.

## Footnotes

1 http://ieg.ou.edu/microarray

2 http://zhoulab5.rccc.ou.edu:8080

## Supplementary Material

FIGURE S1 Average air temperature **(a)** and cumulative precipitation **(b)** over the entire autumn (September to November) in the experiment site. The air temperature and cumulative precipitation were obtained online from the Oklahoma Mesonet Station (Washington Station). Air temperature data were collected at 1.5 m above the soil surface, and precipitation data were collected at the soil surface.

FIGURE S2 Detrended correspondence analysis (DCA) of 16S rRNA gene **(a)** and ITS **(b)** amplicon sequences and GeoChip hybridization **(c)**. Symbols correspond to year of sampling. Colors of the symbols represent sample treatments with filled symbols representing clipped samples and hollow symbols representing control samples. Arrows represent the direction of change under clipping treatment in different years.

FIGURE S3 The composition of bacterial community under clipping and control in 5 years based on 16S rRNA gene sequencing. The phylum *Proteobacteria* was represented by Alpha-, Beta-, Gamma-, and Delta-divisions. The insert table showed the significances of each phylum affected by clipping and sampling year based on ANOVA test. Red indicated *P* < 0.05.

FIGURE S4 The composition of fungal community under clipping and control in 5 years based on ITS sequencing. The insert table showed the significances of each phylum affected by clipping and sampling year based on ANOVA test. Red indicated *P* < 0.05.

FIGURE S5 Constrained ordination analysis of 16S rRNA gene amplicon sequences. **(a)** Canonical correspondence analysis (CCA) of 16S rRNA gene amplicon sequences and environmental variables. **(b)** CCA-based variation partitioning analysis (VPA) of bacterial community structure explained by soil geochemical properties (S), plant diversity (P), climate variables (C), and time (T). Details are described in **Figure 2**.

FIGURE S6 Constrained ordination analysis of ITS amplicon sequences. **(a)** Canonical correspondence analysis (CCA) of ITS amplicon sequences and environmental variables. **(b)** CCA-based variation partitioning analysis (VPA) of fungal community structure explained by soil geochemical properties (S), plant diversity (P), climate variables (C), and time (T). Details are described in **Figure 2**.

FIGURE S7 The relative changes of normalized signal intensities of detected C-degradation genes under clipping in different years. The complexity of C is presented in order from labile to recalcitrant C. Error bars indicate standard error of the mean. Significance is tested by ANOVA as indicated by ^∗∗∗^*P* < 0.001, ^∗∗^*P* < 0.01, ^∗^*P* < 0.05. The full names of the genes in this figure are listed in **Supplementary Table S4**.

FIGURE S8 The relative changes of normalized signal intensities of detected C fixation genes under clipping in different years. Error bars indicate standard error of the mean. Significance is tested by ANOVA as indicated by ^∗∗∗^*P* < 0.001, ^∗∗^*P* < 0.01, ^∗^*P* < 0.05. The full names of the genes in this figure are listed in **Supplementary Table S4**.

FIGURE S9 The relative changes of normalized signal intensities of detected N cycling genes under clipping in different years. Error bars indicate standard error of the mean. Significance is tested by ANOVA as indicated by ^∗∗∗^*P* < 0.001, ^∗∗^*P* < 0.01, ^∗^*P* < 0.05. **(A)**, ammonification; **(B)**, anammox; **(C)**, assimilation; **(D)** assimilatory N reduction; **(E)**, denitrification; **(F)**, dissimilatory N reduction; **(G)**, N assimilation; **(H)**, nitrification; **(I)**, nitrogen fixation. The full names of the genes in this figure are listed in **Supplementary Table S4**.

FIGURE S10 The relative changes of normalized signal intensities of detected P utilization genes under clipping in different years. Error bars indicate standard error of the mean. Significance is tested by ANOVA as indicated by ^∗∗∗^*P* < 0.001, ^∗∗^*P* < 0.01, ^∗^*P* < 0.05. The full names of the genes in this figure are listed in **Supplementary Table S4**.

TABLE S1 Soil geochemical variables, plant biomass and soil respirations under control and clipping in each year.

TABLE S2 The overall microbial community diversity under clipping and control detected by ITS and 16S rRNA gene sequencing data and GeoChip data.

TABLE S3 The significance tests of bacterial and fungal lineages affected by clipping in different years by ANOVA.

TABLE S4 The enzyme/protein encoded by the functional genes shown in **Figures 3, 4**, and **Supplementary Figures S7–S10**.
