# Supplementary figures and images for: Taxonomic and Functional Responses of Soil Microbial Communities to Annual Removal of Aboveground Plant Biomass
Source: Front Microbiol. 2018 May 31;9:954. doi: 10.3389/fmicb.2018.00954 (PMC5990867; doi:10.3389/fmicb.2018.00954)

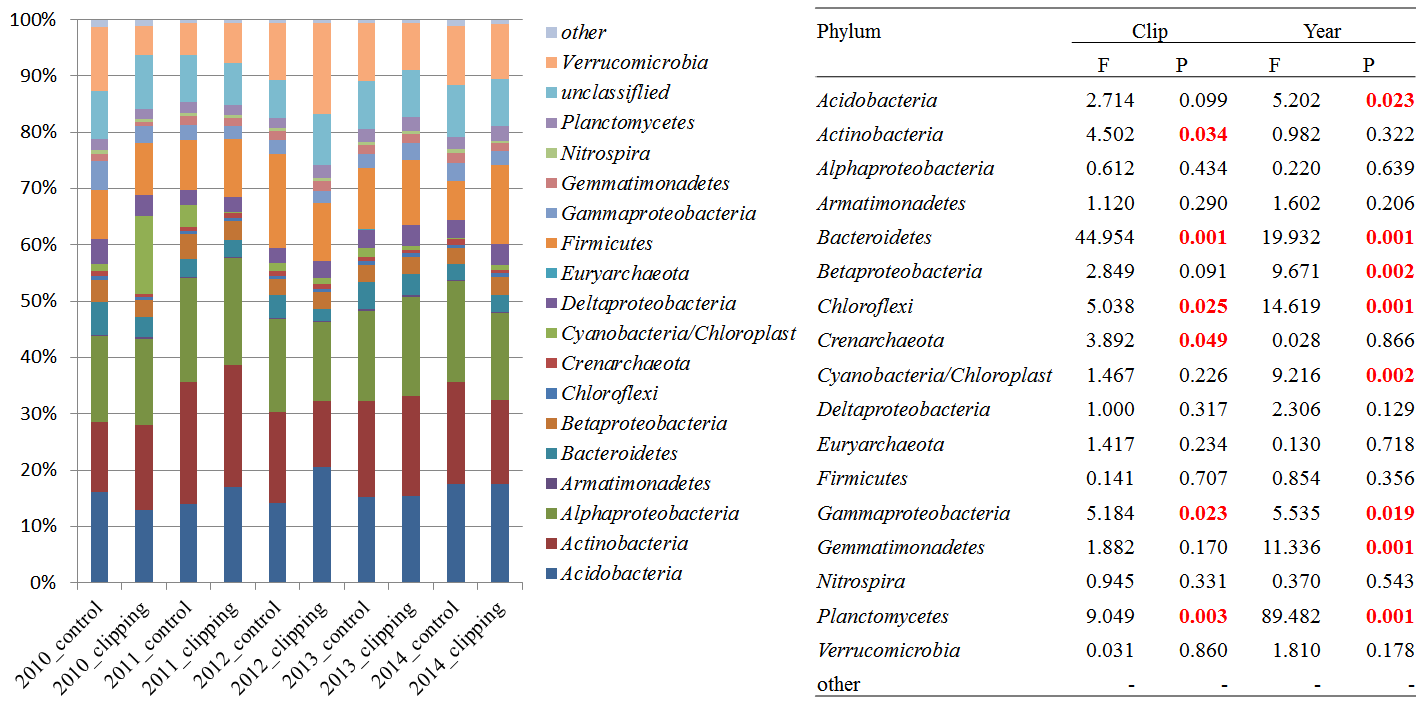

Supplement: FIGURE S3 — The composition of bacterial community under clipping and control in 5 years based on 16S rRNA gene sequencing. The phylum Proteobacteria was represented by Alpha-, Beta-, Gamma-, and Delta-divisions. The insert table showed the significances of each phylum affected by clipping and sampling year based on ANOVA test. Red indicated P < 0.05. [file Image_3.TIF]

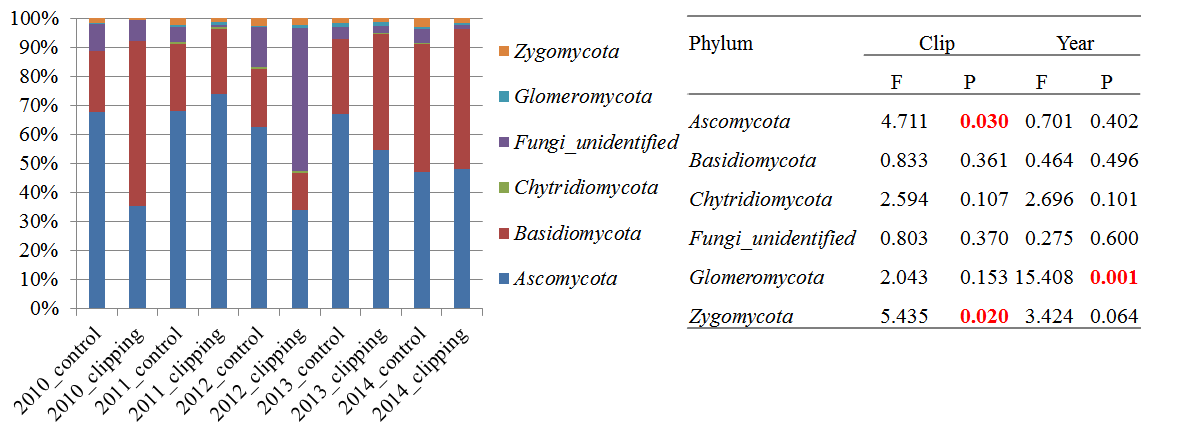

Supplement: FIGURE S4 — The composition of fungal community under clipping and control in 5 years based on ITS sequencing. The insert table showed the significances of each phylum affected by clipping and sampling year based on ANOVA test. Red indicated P < 0.05. [file Image_4.TIF]

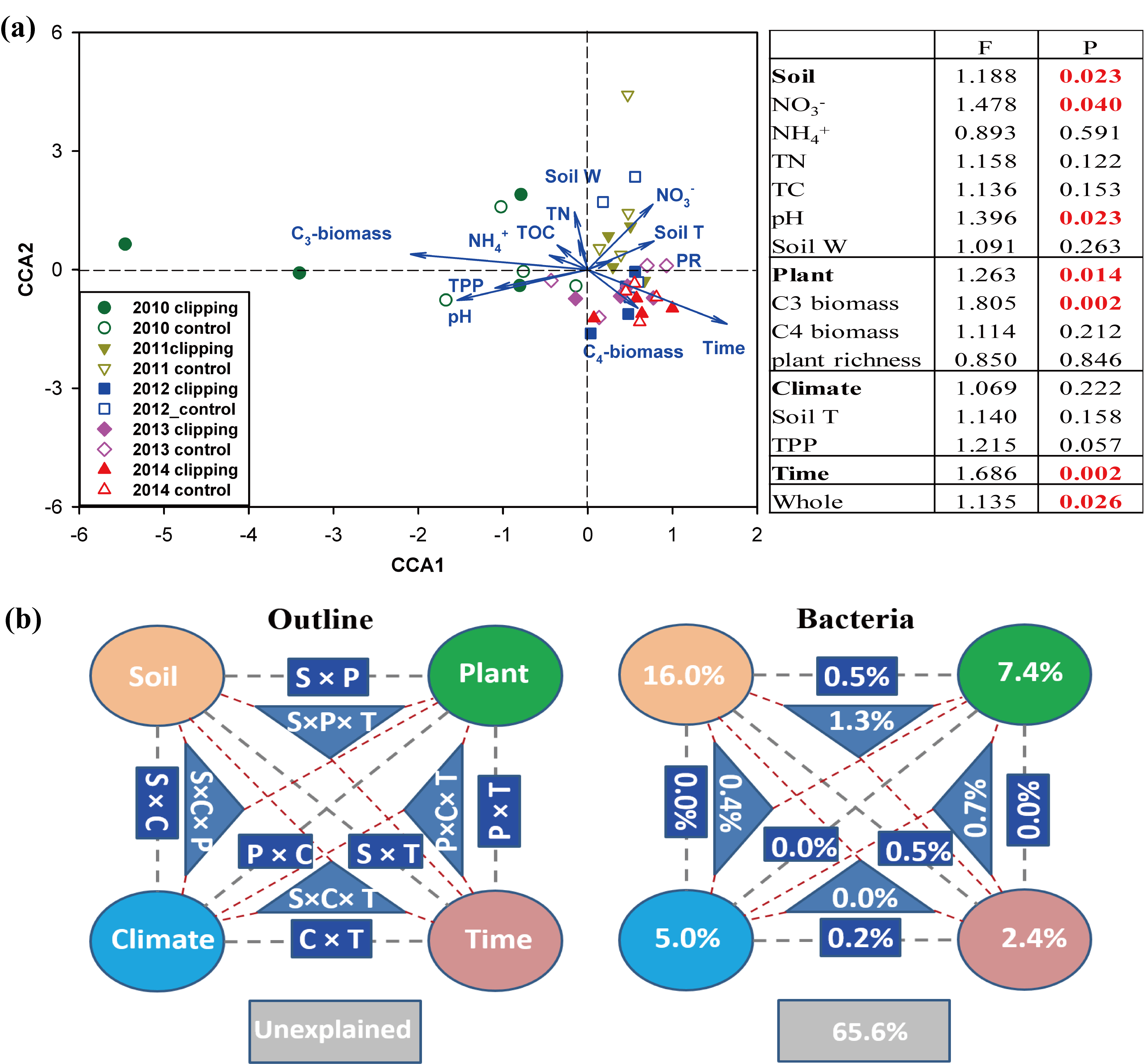

Supplement: FIGURE S5 — Constrained ordination analysis of 16S rRNA gene amplicon sequences. (a) Canonical correspondence analysis (CCA) of 16S rRNA gene amplicon sequences and environmental variables. (b) CCA-based variation partitioning analysis (VPA) of bacterial community structure explained by soil geochemical properties (S), plant diversity (P), climate variables (C), and time (T). Details are described in Figure 2. [file Image_5.TIF]

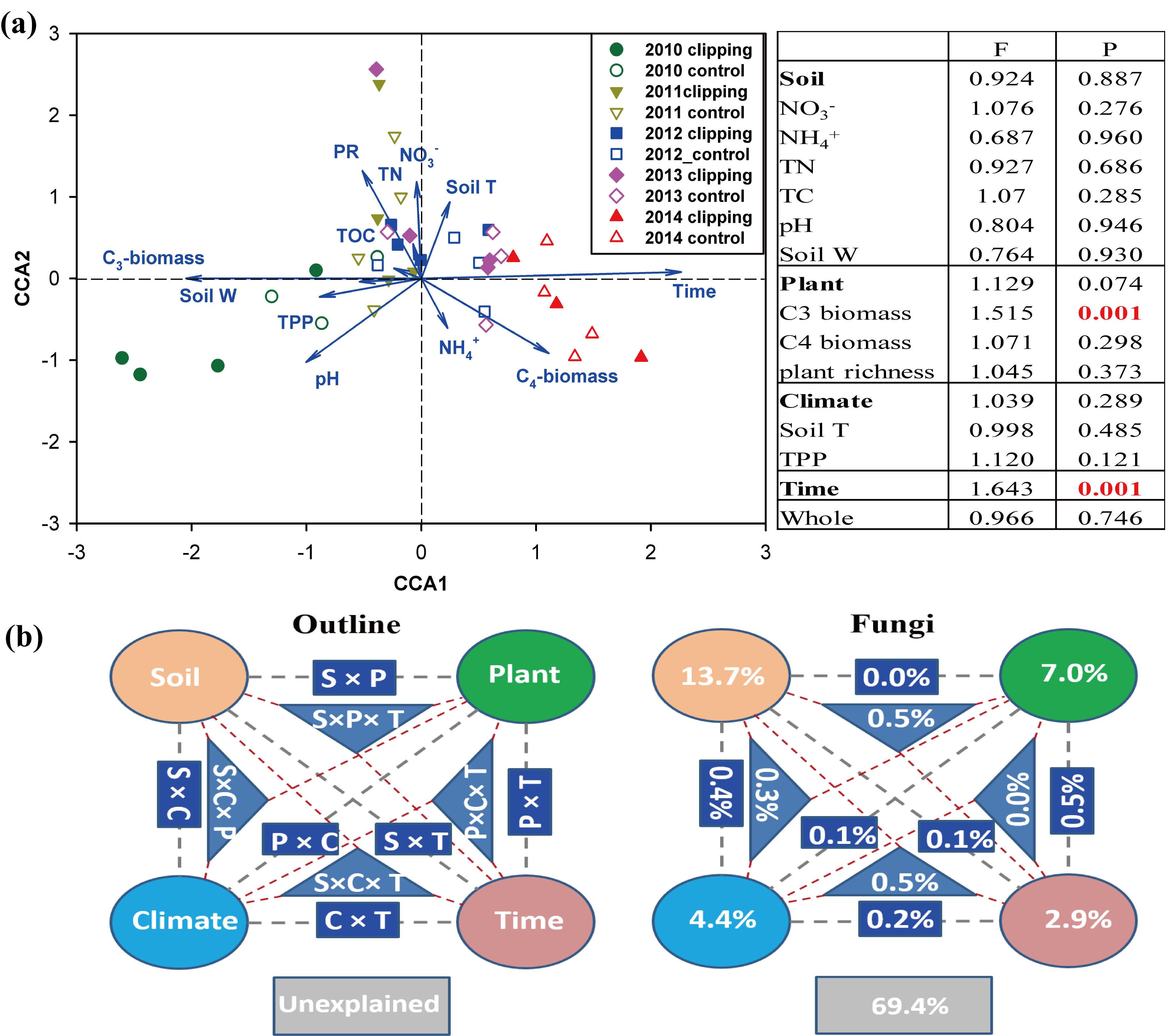

Supplement: FIGURE S6 — Constrained ordination analysis of ITS amplicon sequences. (a) Canonical correspondence analysis (CCA) of ITS amplicon sequences and environmental variables. (b) CCA-based variation partitioning analysis (VPA) of fungal community structure explained by soil geochemical properties (S), plant diversity (P), climate variables (C), and time (T). Details are described in Figure 2. [file Image_6.TIF]
