# Supplementary material for: Taxonomic and Functional Responses of Soil Microbial Communities to Annual Removal of Aboveground Plant Biomass
Source: Front Microbiol. 2018 May 31;9:954. doi: 10.3389/fmicb.2018.00954 (PMC5990867; doi:10.3389/fmicb.2018.00954)
Supplement: TABLE S1 — Soil geochemical variables, plant biomass and soil respirations under control and clipping in each year. [file Table_1.pdf]

Table S1. Soil geochemical variables, plant biomass and soil respirations under control and clipping in each year[1].

	2010		2011		2012		2013		2014	
	Control	Clipping	Control	Clipping	Control	Clipping	Control	Clipping	Control	Clipping
NO_3^--N (mg/kg)	3.02±1.41	2.51±0.42	37.41±12.46	41.33±6.30	22.48±3.43	**7.84±3.71**	3.79±0.94	**1.43±0.38**	13.48±0.36	**2.78±1.31**
NH_4^+-N (mg/kg)	3.32±0.53	3.96±0.36	2.84±0.45	2.96±0.19	4.85±0.78	3.46±0.07	2.99±0.18	3.70±0.58	4.99±1.59	3.43±0.37
TN (%)	0.08±0.01	**0.09±0.01**	0.10±0.02	0.12±0.02	0.11±0.01	0.08±0.01	0.10±0.01	0.11±0.01	0.10±0.01	0.09±0.01
TOC (%)	0.73±0.10	**0.87±0.12**	0.90±0.21	1.13±0.21	1.09±0.21	0.73±0.08	0.90±0.09	1.00±0.17	0.95±0.17	0.78±0.09
Soil pH	6.39±0.09	6.51±0.12	5.98±0.11	5.93±0.06	6.08±0.10	**6.33±0.06**	6.28±0.05	6.27±0.08	5.95±0.06	6.14±0.12
Soil W (m^3 m^{-3})	6.74±0.21	**5.60±0.20**	6.74±0.21	**5.60±0.22**	6.36±1.43	6.20±1.68	4.32±0.89	3.84±0.45	3.54±0.64	4.56±0.61
Soil T (°C)	16.16±0.21	**16.84±0.21**	18.29±0.25	**18.91±0.26**	18.53±0.22	**18.73±0.21**	16.43±0.20	**16.63±0.19**	17.25±0.10	**17.65±0.10**
C_3 biomass (g m^{-2})	451±57	448±96	180±12	157±22	234±35	294±46	106±45	283±89	20±7	6±9
C_4 biomass (g m^{-2})	110±25	**36±13**	72±3	82±14	49±44	50±37	57±25	100±40	107±23	**196±31**
Total biomass (g m^{-2})	561±39	484±89	252±10	239±8	283±20	344±69	163±35	**384±66**	128±26	**203±36**
Plant richness	4.50±1.32	5.50±0.86	6.00±0.58	4.75±0.48	3.50±0.50	**6.00±0.57**	5.00±0.71	6.00±1.08	3.75±0.48	4.75±0.85
HR (μmol m^{-2} s^{-1})	2.00±0.19	2.22±0.20	0.91±0.17	0.58±0.16	0.79±0.08	0.90±0.11	1.41±0.21	**1.13±0.14**	1.35±0.21	**1.9±0.42**
AR (μmol m^{-2} s^{-1})	1.23±0.18	**1.92±0.27**	2.18±0.51	3.36±0.94	2.13±0.19	2.00±0.23	1.71±0.25	1.92±0.26	0.34±0.15	0.11±0.25
TR (μmol m^{-2} s^{-1})	3.23±0.27	**4.14±0.31**	3.10±0.52	3.96±0.94	2.92±0.23	2.90±0.27	3.12±0.30	3.04±0.33	1.69±0.24	**1.90±0.42**

[1] The variables were compared between clipping and control in each year. Those significantly (*P* < 0.05) changed were indicated in boldface. NO_3^--N: soil nitrate nitrogen; NH_4^+-N: soil ammonium nitrogen; TN: soil total nitrogen; TOC: soil total organic carbon; Soil W: soil water content; Soil T: soil temperature; HR: soil heterotrophic respiration; AR: soil autotrophic respiration; TR: soil total respiration.
