# Supplementary material for: Taxonomic and Functional Responses of Soil Microbial Communities to Annual Removal of Aboveground Plant Biomass
Source: Front Microbiol. 2018 May 31;9:954. doi: 10.3389/fmicb.2018.00954 (PMC5990867; doi:10.3389/fmicb.2018.00954)
Supplement: TABLE S2 — The overall microbial community diversity under clipping and control detected by ITS and 16S rRNA gene sequencing data and GeoChip data. [file Table_2.pdf]

Table S2. The overall microbial community diversity under clipping and control detected by ITS and 16S rRNA gene sequencing data and GeoChip data[1].

	RichnessOTU	Shannon index	Simpson index	Evenness
16S				
2010 Clipping	5753±288	6.86±0.26	131.96±85.94	0.79±0.03
2010 Control	6350±204	7.41±0.13	283.00±57.90	0.85±0.01
2011 Clipping	5363±182	7.21±0.12	324.31±94.64	0.84±0.01
2011 Control	5349±223	7.09±0.19	262.48±72.74	0.83±0.02
2012 Clipping	4888±221*	6.80±0.15	137.40±47.31	0.80±0.01
2012 Control	5120±207	6.94±0.09	136.86±31.05	0.81±0.01
2013 Clipping	5922±298	7.41±0.12	356.88±80.99	0.85±0.01
2013 Control	5971±260	7.29±0.17	252.43±85.99	0.84±0.02
2014 Clipping	5588±441	7.18±0.28	270.11±76.96	0.83±0.03
2014 Control	5429±114	7.27±0.02	293.48±26.63	0.85±0.01
ITS				
2010 Clipping	263±75	2.30±0.65	7.55±4.76	0.41±0.09
2010 Control	390±51	3.49±0.28	12.80±2.68	0.59±0.04
2011 Clipping	383±52	3.45±0.51	14.67±4.81	0.58±0.08
2011 Control	394±49	3.43±0.31	11.19±2.87	0.5±0.04
2012 Clipping	328±42	2.69±0.42	6.83±3.69	0.46±0.06
2012 Control	345±63	3.41±0.27	11.50±1.59	0.59±0.03
2013 Clipping	409±48	3.39±0.38	13.46±4.61	0.56±0.06
2013 Control	392±42	3.37±0.39	13.06±5.35	0.56±0.06
2014 Clipping	279±38	2.53±0.59	7.02±6.49	0.44±0.09
2014 Control	386±28	3.19±0.36	9.40±6.90	0.53±0.06
GeoChip				
2010 Clipping	31291±1365*	9.24±0.05*	3187.05±180.98*	0.89±0.001
2010 Control	34436±793	9.32±0.03	3467.50±192.58	0.89±0.001
2011 Clipping	39967±274*	9.51±0.01	4494.90±69.90	0.90±0.001
2011 Control	36617±1119	9.42±0.05	4122.34±263.59	0.90±0.002
2012 Clipping	29176±913*	9.32±0.02*	3210.06±85.35*	0.91±0.002*
2012 Control	38344±834	9.49±0.01	4093.37±60.17	0.90±0.001
2013 Clipping	41008±962#	9.54±0.02	4365.27±138.28#	0.90±0.0004
2013 Control	39518±484	9.51±0.02	4170.67±98.20	0.90±0.001
2014 Clipping	3634±688*	9.46±0.02#	4072.88±107.60	0.90±0.001
2014 Control	34642±765	9.42±0.02	3851.06±91.37	0.90±0.001

[1] The differences between clipping and control in each year were tested by two-tailed paired *t*-tests, indicated by * when $P < 0.05$ or # when $P < 0.10$.
