# Supplementary material for: Taxonomic and Functional Responses of Soil Microbial Communities to Annual Removal of Aboveground Plant Biomass
Source: Front Microbiol. 2018 May 31;9:954. doi: 10.3389/fmicb.2018.00954 (PMC5990867; doi:10.3389/fmicb.2018.00954)
Supplement: TABLE S3 — The significance tests of bacterial and fungal lineages affected by clipping in different years by ANOVA. [file Table_3.pdf]

Table S3. The significance tests of bacterial and fungal lineages affected by clipping in different years by ANOVA[1].

	2010	2011	2012	2013	2014
**Bacteria**					
Acidobacteria	2***	2**	3***	1	2
Actinobacteria	11***	6	16***	3	4***
Alphaproteobacteria	13	1	15***	7**	9***
Armatimonadetes	0	0	0	2*	0*
Bacteroidetes	3***	0	9***	2*	2
Betaproteobacteria	1***	3	4	0	3
Chloroflexi	2**	0**	0*	1	0*
Crenarchaeota	0**	0	0	0	0*
CyanobacteriaChloroplast	1*	1	1	1	2
Deltaproteobacteria	3**	2	2**	0**	5**
Euryarchaeota	0	0	0	0	0
Firmicutes	6	1	3**	1	6*
Gammaproteobacteria	2*	0	3	1*	2*
Gemmatimonadetes	0*	0	0	0	0*
Nitrospira	0	0	0	0	1**
Planctomycetes	3***	1	0***	0	3***
Verrucomicrobia	4***	1*	1*	0	1
**Fungi**					
Ascomycota	6**	2	6**	3	6
Basidiomycota	0	1	1	1	1
Chytridiomycota	1	0	1	2	1*
Fungi_unidentified	0	0	1*	0	0
Glomeromycota	2***	0	1***	0	2*
Zygomycota	0**	1	0	0	1*

[1] The number indicated the significantly ($P < 0.05$) and marginally significantly ($P < 0.10$) changed genera under clipping. The significance of different phyla was represented by *** when $P < 0.001$, ** when $P < 0.01$ and * when $P < 0.05$.
