# Supplementary material for: Taxonomic and Functional Responses of Soil Microbial Communities to Annual Removal of Aboveground Plant Biomass
Source: Front Microbiol. 2018 May 31;9:954. doi: 10.3389/fmicb.2018.00954 (PMC5990867; doi:10.3389/fmicb.2018.00954)
Supplement: TABLE S4 — The enzyme/protein encoded by the functional genes shown in Figures 3, 4, and Supplementary Figures S7–S10. [file Table_4.pdf]

Table S4. The enzyme/protein encoded by the functional genes shown in figure 3, 4, S7, S8, S9 and S10.

Gene category	Subcategory	Gene/enzyme name	Phylogenetic group	Enzyme/ protein encoded
C fixation	Calvin cycle	Rubisco	Bacteria	Ribulose bisphosphate carboxylase
	Reductive acetyl-CoA pathway	CODH	Bacteria	Carbon monoxide dehydrogenase
	3-hydroxypropionate cycle	PCC	Bacteria	Propionyl-CoA carboxylase
	Reverse tricarboxylic acid cycle	*aclB*	Bacteria	ATP citrate lyase beta subunit
C degradation	Starch	glucoamylase	Bacteria/Fungi	Glucoamylase
	Starch	*cda*	Bacteria	Cyclomaltodextrinase
	Starch	*amyA*	Bacteria	Alpha-amylase
	Starch	*amyx*	Bacteria	Pullulanase
	Starch	*nplT*	Bacteria	Neopullulanase
	Starch	*apu*	Bacteria	Amylopullulanase
	Starch	isopullulanase	Bacteria	Isopullulanase
	Starch	*pulA*	Bacteria	Pullulanase
	Inulin	inulinase	Bacteria/Fungi	Inulinase
	Hemicellulose	xylanase	Bacteria	Xylanase
	Hemicellulose	mannanase	Bacteria/Fungi	Mannanase
	Hemicellulose	*xylA*	Bacteria	Xylose isomerase
	Hemicellulose	xylose isomerase	Oomycetes	Xylose isomerase
	Hemicellulose	*ara*	Bacteria/Fungi	Arabinofuranosidase
	Lactose	lactase	Fungi	Lactase
	Pectin	pectinase	Bacteria	Pectinase
	Pectin	pectin lyase	Oomycetes	Pectin lyase
	Pectin	*pg*	Oomycetes	Polygalacturonase
	Pectin	*pme*	Fungi	Pectin methylesterase
	Pectin	*pec*	Fungi	Pectate lyase
	Pectin	*rgh*	Bacteria/Fungi	rhamnogalacturonase
	Pectin	*pme*	Bacteria	Pectinesterase
	Pectin	exopolygalacturonase	Fungi	Exopolygalacturonase
	Pectin	*rgaE*	Bacteria/Fungi	lipolytic enzyme
	Pectin	*rgl*	Bacteria/Fungi	Polysaccharide lyase
	Pectin	pectate lyase	Oomycetes	Pectate lyase
	Pectin	endopolygalacturonase	Fungi	Endopolygalacturonase
	Cellulose	*axe*	Fungi	Acetyl xylan esterase
	Cellulose	cellobiase	Bacteria/Fungi	Cellobiase
	Cellulose	endoglucanase	Bacteria/Fungi	Endoglucanase
	Cellulose	cellulase	Parabasalia	Cellulase
	Cellulose	exoglucanase	Bacteria/Fungi	Exoglucanase
	Phospholipids	phospholipase C	Fungi	Phospholipase subunit C
	Phospholipids	phospholipase A2	Fungi	Phospholipase subunit A2
	Phospholipids	phospholipase D	Fungi	Phospholipase subunit D
	Camphor	camdcab	Bacteria	Camphor 5-monooxygenase
	Terpenes	*lmeH*	Bacteria	Limonene-1,2-epoxide hydrolase
	Terpenes	*lmo*	Bacteria	Limonene 1,2-monooxygenase

	Terpenes	*cdh*	Bacteria	Carveol dehydrogenase
	Cutin	cutinase	Bacteria/Fungi	Cutinase
	Tannins	tannase	Bacteria	
	Chitin	acetylglucosaminidase	Bacteria/Fungi	Acetylglucosaminidase
	Chitin	chitin deacetylase	Fungi	Chitin deacetylase
	Chitin	chitinase	Bacteria/Fungi	Chitinase
	Vanillin/Lignin	*vanA*	Bacteria	Vanillate monooxygenase
	Vanillin/Lignin	*vdh*	Bacteria/Fungi	Vanillin dehydrogenase
	Vanillin/Lignin	Phenol oxidase	Bacteria/Fungi	Phenol oxidase
	Vanillin/Lignin	Ligninase	Fungi	Ligninase
	Vanillin/Lignin	*glx*	Bacteria/Fungi	glyoxal oxidase
	Vanillin/Lignin	*mnp*	Fungi	Manganese peroxidase
N cycling	Ammonification	*gdh*	Bacteria/Fungi	Glutamate dehydrogenase
	Ammonification	*glnA*	Fungi	Glutamine synthetase
	Ammonification	*ureC*	Bacteria	Urease
	Anammox	*hzsA*	Bacteria	Hydrazine synthase
	Anammox	*hzo*	Bacteria	Hydroxylamine oxidoreductase
	Assimilation	Glutamine synthetase	Protists	Glutamine synthetase
	Assimilation	nitrate transporter	Bacteria	Nitrate transporter
	Assimilation	glutamate synthase	Protists	Glutamate synthase
	Assimilation	nitrite reductase	Protists	Nitrite reductase
	Assimilation	nitrite transporter	algae	Nitrite transporter
	Assimilation	ammonium transporter	algae	Ammonium transporter
	Assimilatory N reduction	*narB*	Bacteria	Nitrate reductase
	Assimilatory N reduction	*nir*	Bacteria	Nitrite reductase
	Assimilatory N reduction	*nirA*	Bacteria	Ferredoxin-nitrite reductase
	Assimilatory N reduction	*nirB*	Bacteria	NADPH-nitrite reductase
	Assimilatory N reduction	*nasA*	Bacteria	Nitrate reductase
	Denitrification	*norB*	Bacteria	Nitric-oxide reductase
	Denitrification	*nirK*	Bacteria	Nitrite reductase
	Denitrification	*nirS*	Bacteria	Nitrite reductase
	Denitrification	*cnorB*	Bacteria	Nitric oxide reductase subunit B
	Denitrification	*nosZ*	Bacteria	Nitrous-oxide reductase
	Denitrification	*narG*	Bacteria	Membrane bound nitrate reductase
	Dissimilatory N reduction	*nrfA*	Bacteria	Nitrite reductase subunit cytochrome c552
	Dissimilatory N reduction	*napA*	Bacteria	Periplasmic nitrate reductase
	N Assimilation	Nitrate reductase	Bacteria	Nitrate reductase
	Nitrification	*amoA*	Bacteria	Ammonia monooxygenase
	Nitrification	*hao*	Bacteria	Hydroxylamine oxidoreductase
	Nitrogen fixation	*nifH*	Bacteria	nitrogenase
	Phosphorus oxidation	*htxA*	Bacteria	Phytanoyl-CoA dioxygenase
	Phosphorus oxidation	*ptxD*	Bacteria	Phosphonate dehydrogenase
	Phytic acid hydrolysis	Phytase	Bacteria/Fungi	Phytase
	Polyphosphate degradation	*ppk2*	Bacteria	Polyphosphate kinase 2

P utilization	Polyphosphate degradation	*ppn*	Fungi	Endopolyphosphatase
	Polyphosphate degradation	*ppx*	Bacteria	Exopolyphosphatase
	Polyphosphate synthesis	*ppk*	Bacteria	Polyphosphate kinase
